# The learning environment in the obstetrics and gynecology clerkship: an exploratory study of students' perceptions before and after the clerkship

**DOI:** 10.3402/meo.v20.27273

**Published:** 2015-06-15

**Authors:** Laura E. Baecher-Lind, Katherine Chang, Maria A. Blanco

**Affiliations:** 1 Department of Obstetrics and Gynecology, Tufts University School of Medicine, Boston, MA, USA; 2 Department of Obstetrics and Gynecology, McGaw Medical Center of Northwestern University, Chicago, IL, USA; 3 Office of Educational Affairs, Tufts University School of Medicine, Boston, MA, USA

**Keywords:** student mistreatment, workplace learning, Ob/Gyn clerkship

## Abstract

For reasons that remain not entirely clear, Obstetrics and Gynecology (Ob/Gyn) clerkships often exhibit comparatively higher rates of medical student mistreatment. To explore perceptions of our local learning environment, focus groups were held with students yet to start (pre-students) and students having completed (post-students) their Ob/Gyn clerkship. Topics of discussion included learning expectations and experiences, perceptions of mistreatment, and suggestions for improving the learning environment and student treatment. Using a naturalistic approach, we conducted a conventional content analysis to identify emergent themes. Nine pre-students and nine post-students participated. While pre-students anticipated being actively engaged, they also expected – based on peer accounts – to be subject to an unwelcoming learning environment on the Ob/Gyn clerkship, despite working hard to become team members. Due to patient advocacy and protection concerns, post-students reported low levels of student involvement and, subsequently, an overall passive learning experience. Students from both groups offered valuable suggestions for improving the learning environment and student treatment. The sensitive nature of Ob/Gyn clinical encounters may lead to overprotective behaviors that contribute to students feeling mistreated and excluded from patient care and team membership. Students’ experiences during Ob/Gyn clerkships could be improved by better balancing patient advocacy and student involvement. Practical implications to address these issues are offered.

Nearly 20% of graduating medical students report experiencing at least one incident of mistreatment during medical school, ranging from incidents of sexual assault to more nebulous incidents such as humiliation or belittling (1–3). In addition to potentially decreasing students’ interest in a given specialty (4), students may perceive issues emblematic of a poor learning environment – such as disinterested faculty educators, inadequate student supervision, or excessive downtime during a clerkship – as forms of mistreatment (5). Nested within the ‘hidden curriculum’ (4), eradicating or even reducing mistreatment is difficult – since students occupy the lowest tier of the medical hierarchy where behaviors no longer accepted are perpetuated.

The Obstetrics and Gynecology (Ob/Gyn) clerkship is frequently associated with high rates of student mistreatment, with approximately 25% of students reporting at least one mistreatment episode during their Ob/Gyn clerkship (2). Studies have shown that high stress and high stakes clinical environments – such as those commonly found at the Ob/Gyn workplace – contribute to higher levels of disrespect and communication breakdown (6, 7). To further explore this area, we conducted a qualitative descriptive study using focus groups of third- and fourth-year medical students to explore concepts related to expectations and perceptions of the learning environment and student treatment on the Ob/Gyn clerkship.

At our school, approximately 200 students per academic year complete their Ob/Gyn clerkship at one of six affiliated clinical sites. Most students spend 6 weeks at their Ob/Gyn site, except for approximately 36 students who are enrolled in the Longitudinal Integrated Clerkship (LIC). Nearly half of the students complete their clerkship at our main academic medical center, and the other one-half complete their clerkship at an affiliated site. All six clinical sites share the same policies, procedures, learning objectives, and evaluation formats – and students report equivalent volume and breadth of clinical experiences. At the start of this study, end-of-course evaluations revealed that nearly 20% of students completing their 6-week Ob/Gyn clerkship experienced at least one episode of mistreatment.

## Methods

All third- and fourth-year medical students were invited to participate in the focus groups. Enrollment was restricted to third-year students who had not started their Ob/Gyn clerkship, to third- and fourth-year students who completed their Ob/Gyn clerkship, and to non-LIC students. Students were offered free lunch and a $20 gift card for their participation. Focus groups were limited to six students per group; the first six students who signed up to participate in each focus group were enrolled in the study (8). The study was approved by the Institutional Review Board of Tufts Health Sciences.

## Data collection

Four 90-min focus groups were conducted from July to August 2013: Two with pre-clerkship students and two with post-clerkship students, and the possibility of additional groups if methodologically warranted (9). The focus groups were conducted by MB, a formally-trained educator with no direct role in teaching and/or evaluating students during the Ob/Gyn clerkship. Focus groups topics included students’ assessments of the clerkship learning experience, perceptions of treatment, and suggestions for improving both.

Focus groups discussions were audio-recorded and transcribed by KC with participants’ identities concealed. Using a naturalistic approach, a conventional content analysis was conducted (10). All three authors independently conducted an initial open coding to identify distinct concepts and categories, followed by axial coding to confirm and explore relationships among them (9). A separate, iterative coding was conducted by LB using Atlas.ti7. The authors then met to systematically examine convergence and divergence of themes, discuss discrepancies, and reach consensus. Emergent themes were then compared with those discussed in the literature related to student mistreatment.

## Results

Eighteen subjects (nine pre- and nine post-clerkship students) who had rotated through or were scheduled to rotate through four different sites participated in the focus groups. The following findings are presented according to the guiding research questions.

### Expectations and perceptions of the learning environment

I expect to be treated as a part of the team that doesn't know much but wants to learn. (Pre-clerkship student)

They should advocate and say the students are a part of the healthcare team. (Post-clerkship student)

Being actively engaged in the learning process, given appropriate clinical oversight, and valued as team members in helping to support patient care were expectations pre-clerkship students reported having for all clerkships. However, they also anticipated ‘pushback’ from staff and having to work ‘twice as hard’ to earn team respect and membership in the Ob/Gyn clerkship. They noted that Ob/Gyn is female-dominated, as opposed to surgery, and poses challenges due to the intimate and sensitive nature of the clinical encounters. That is, students anticipated and accepted occasional exclusion from patient care in situations involving abortion or miscarriage, or in respect of patients’ culture or religious practices. Most pre-clerkship students agreed that treatment expectations were affected by ‘what you hear’ and ‘stories from’ other students.

Post-clerkship students emphasized feeling ignored during the Ob/Gyn clerkship, ‘in the way’, and not valued as members of the medical team. They voiced poor educational advocacy by faculty, residents, and nurses, which limited the value of their clinical experience, and felt such exclusions constituted missed opportunities for practicing pelvic examination skills, participating in childbirth, or observing/assisting with surgical procedures. Students described an overall passive learning experience throughout the Ob/Gyn clerkship, and much ‘wasted time’ spent waiting for patient care opportunities to arise. Some cited a culture of disrespect – with staff ‘protecting’ patients from student involvement. Students described an especially strong ethic of patient advocacy during childbirth-related experiences that extended to ED consultations and outpatient office visits. According to post-clerkship students, the greatest barrier to patient care and learning stemmed from an overprotective ethos among providers – not from patients’ preferences or demands.

### Perceptions of student treatment

[For me, mistreatment are things like] sexual harassment. Being forced to watch patients be mistreated, whether it's physical, psychological, or emotional, and not being able to do anything about it. (Pre-clerkship student)

They should at least treat you like a stranger. You would not even treat a stranger that poorly. They should at least treat you like a person. (Post-clerkship student)

Pre-clerkship students believed they should be treated with respect and tolerance as novice clinical trainees, and that mistreatment encompassed a wide range of episodic behaviors ranging from the concrete (e.g., sexual/physical assault, performing personal errands) to the less obvious (e.g., lack of dedication to teaching). Many attributed student mistreatment to the hierarchical structure of medicine, a stressful work environment, and personality clashes – noting that ‘mistreatment feeds or breeds more mistreatment’. When asked about reporting such behaviors, pre-clerkship students preferred to ‘talk about it first’ and ensure it was ‘consistent and personal’. They also acknowledged the role of individual student's personality.

Post-clerkship students’ perceptions of mistreatment focused on a lack of respect, including ignoring the student and wasting their time. They attributed mistreatment to the sensitive issues involved in Ob/Gyn care and an atmosphere of excessive patient protectiveness. These students stated that mistreatment warranting report had to be something that affected personal safety or was ‘completely out there’. They, too, pointed out that reporting mistreatment was very ‘student-specific’ in terms of both perceptions of mistreatment and comfort in reporting.

### Suggestions for improving the learning environment and student treatment

I think it's important that expectations are clearly laid out before the rotation starts. (Pre-clerkship student)

I think they should advocate and say the students are a part of the healthcare team. (Post-clerkship student)

Both pre- and post-clerkship students suggested ways to improve the learning environment and student treatment during the Ob/Gyn clerkship, including: 1) clarifying roles and expectations; 2) enhancing communication; and 3) introducing them to patients as team members to encourage patients’ acceptance. Other suggestions included open and ongoing communication with the clerkship director (pre-clerkship students) and greater opportunity for active participation in patient care (post-clerkship students).

## Discussion

In addition to general clerkship expectations, pre-clerkship students were concerned with hearsay of a hostile and unsupportive learning environment on the Ob/Gyn clerkship. Since research has shown individuals’ expectations to strongly correlate with their actual experiences (11–13), strong anticipations may color subsequent perceptions accordingly. Medical students having completed the Ob/Gyn clerkship describe a passive learning environment whereby they felt isolated, de-valued, and unnecessarily excluded from clinical activities.

Functioning as a team is a necessary part of medicine, and students are keenly aware of their roles and value as participating members. Rejection by patients or medical team members represents a ‘professional lapse’ that students must process and negotiate (14). Similarly, the resulting ‘downtime’ of missed learning opportunities, coupled with faculty disinterest in teaching, contributes to perceptions of a suboptimal learning environment as mistreatment.

Post-clerkship comments suggested that the Ob/Gyn learning environment at some sites violates behavioral tenants relevant to workplace learning, such as the importance of setting clear expectations, providing opportunities for supervised practice (15), and nurturing a ‘curriculum for the workplace’ to promote trainees’ learning and professional development (16) through actively coaching, modeling, and questioning trainees.

Although some factors contributing to students’ perceptions of mistreatment (e.g., suboptimal learning environments) pertain to clerkships in general, the influence of providers’ patient advocacy and protection on student engagement may be unique to Ob/Gyn. Although caregivers may assume they are serving their patients’ best interests by limiting student involvement, recent data indicate that patients value student involvement to a greater degree than supervisors may realize (17).

Our study has several limitations. First, students’ participation was voluntary, which may have resulted in a self-selected sample of students with an interest in the topic. Second, as with any focus group, participants may have been prone to ‘group think’. Lastly, although data saturation was achieved, our sample was small and drawn from a single institution over a relatively short time period – all of which may limit the generalizability of our findings.

### Educational implications

Based on these study findings, we implemented the following strategies to enhance the learning environment (and reduce perceived student mistreatment) in our Ob/Gyn clerkship:

- Formal introductions: We created a ‘face sheet’ of the students rotating through Labor and Delivery so nurses know students who will be on the unit each week. For students, we included a similar listing of all faculty, fellows, residents, and nurses in the online student orientation manual.
- Nurse shadowing program: Early in the clerkship, students work alongside a senior labor and delivery nurse to admit patients, start IVs, and perform Apgar scoring.
- Semiannual teaching evaluations with ‘teaching tips’: Each Ob/Gyn faculty member and resident receives a: 1) ‘report card’ with student comments from the end-of-clerkship evaluations; 2) summary of these comments to facilitate formative improvement; and 3) ‘teaching tip’ derived from students’ comments and relevant educational literature. These ‘report cards’ are also sent to the department chair (for faculty) and program director (for residents) and are subject to annual reviews.
- Education chief resident: A formal position for a PGY-4 Ob/Gyn resident was created to serve as a medical student liaison during the clerkship – meeting with students during the orientation session and participating in the mid- and end-of-clerkship feedback sessions. The education chief resident is selected among all fourth-year residents based on demonstrated commitment to medical education and is provided financial support for a medical education research project and participation in an educational conference.
